# An Intergenic rs9275596 Polymorphism on Chr. 6p21 Is Associated with Multiple Sclerosis in Latvians

**DOI:** 10.3390/medicina56040154

**Published:** 2020-03-31

**Authors:** Natalia Paramonova, Ilva Trapina, Kristine Dokane, Jolanta Kalnina, Tatjana Sjakste, Nikolajs Sjakste

**Affiliations:** 1 Genomics and Bioinformatics, Institute of Biology of the University of Latvia, LV-1004 Riga, Latvia; natalia.paramonova@lu.lv (N.P.); kristine.dokane@lu.lv (K.D.); jolanta@ljmc.lv (J.K.); Tatjana.Sjakste@lu.lv (T.S.); nikolajs.sjakste@lu.lv (N.S.); 2 Department of Medical Biochemistry of the University of Latvia, LV-1004 Riga, Latvia

**Keywords:** rs9275596, the major histocompatibility complex (MHC), Human leukocyte antigen (HLA), autoimmune diseases, multiple sclerosis

## Abstract

*Background and objectives:* Multiple sclerosis (MS) is a chronic inflammatory disease of the central nervous system, leading to demyelination of neurons and potentially debilitating physical and mental symptoms. The disease is more prevalent in women than in men. The major histocompatibility complex (MHC) region has been identified as a major genetic determinant for autoimmune diseases, and its role in some neurological disorders including MS was evaluated. An intergenic single-nucleotide polymorphism (SNP), rs9275596, located between the *HLA-DQB1* and *HLA-DQA2* genes, is in significant association with various autoimmune diseases according to genome-wide association studies (GWASs). A cumulative effect of this SNP with other polymorphisms from this region was revealed. The aim of the study was to verify the data on rs9275596 association in multiple sclerosis in a case/control study of the Latvian population and to evaluate eventual functional significance of allele substitutions. *Materials and Methods:* rs9275596 (chr6:32713854; GRCh38.p12) was genotyped in 273 MS patients and 208 controls on main and sex-specific associations. Eventual functional significance of allele substitutions was evaluated in silico using publicly available tools. *Results:* The rs9275596 rare alleles were identified as a disease susceptibility factor in association with the MS main group and in affected females (*p* < 0.001 and *p* < 0.01, respectively). Risk factor genotypes with rare alleles included were associated with the MS common cohort (*p* < 0.002) and female cohort (odds ratio, OR = 2.24) and were identified as disease susceptible in males (OR = 2.41). It was shown that structural changes of rs9275596 affect the secondary structure of DNA. Functional significance of allele substitutions was evaluated on the eventual sequence affinity to transcription factors (TFs) and splicing signals similarity. A possible impact of the particular polymorphisms on the transcription and splicing efficiency is discussed. *Conclusions:* Our results suggest susceptibility of rs9275596 to multiple sclerosis in Latvians.

## 1. Introduction

Multiple sclerosis (MS) is a chronic inflammatory, demyelinating disease of the central nervous system, affecting about 2.5 million people around the world with greater prevalence in the northern hemisphere (0.5–1.5 per 1,000,000) [1]. This disease tends to be more prevalent in women than in men [2,3]. The disease develops in genetically susceptible individuals with contributions of environmental factors, such as vitamin D deficiency, sunlight exposure, and infection [4,5]. MS-susceptible loci have been found in regions containing genes involved in particular pathways of importance to T-cell differentiation [6], with immune, co-stimulatory, and signal transduction functions [7,8,9,10,11,12].

The human leukocyte antigen (HLA) allele locus is a region of about 4 Mbps located on the short arm of chromosome 6 (Chr.6p21.3), also known as the region of the major histocompatibility complex (MHC) location. Molecules encoded in this region are involved in the innate and adaptive immune responses, antigen presentation, and inflammation regulation [13]. The MHC has been identified as a major genetic determinant for various autoimmune diseases [14,15,16]. Its role in susceptibility to some neurological disorders was recently revealed [17,18]. Some studies report the influence of the *HLA-DRB1* alleles on the genetic susceptibility to MS and their impact on disability progression [19]; HLA-DRB1*1501 has been shown to increase disease severity in MS [20].

The strongest association in the combined cohort of Chinese and European ancestry of the GWAS [15] was found for rs9275596 (odds ratio, OR = 0.63, *p* = 1.6 × 10^−26^) located in the MHC, a ~170 kb intergenic region that includes the HLA-DRB1, -DQA1 and -DQB1 genes. This polymorphism achieves genome-wide significance in each investigated cohort. It was previously found that these gene loci are associated with risk of chronic hepatitis B infection [21] and systemic sclerosis stratified for anti-DNA topoisomerase I or anticentromere autoantibodies [22].

The coexistence of autoimmune diseases recently evaluated statistically [23] suggests the possibility of their common origin. The Chr. 6p21 region confers the most important and best-documented risk factor for MS (refer to GWAS data) [11]. It appears that there is a large potential for the above-mentioned Chr. 6p21 region association studies to provide novel insights to the MS pathogenesis in every particular human population. This assumption motivated our further investigation and determined the aim of the current study, to genotype the rs9275596 polymorphism (6:32713854) on the MS main and sex-specific association in the Latvian population and to evaluate this SNP for the eventual functional significance of allele substitutions in silico.

## 2. Materials and Methods

### 2.1. Case-Control Study

The case group consisted of 273 MS patients (average age: 42.42 ± 11.31 years) referred to the Latvian Maritime Medicine Centre, Vecmilgravis Hospital. MS patients were diagnosed according to the revised 2010 McDonald criteria [24] and assigned to relapse-remitting MS course (191 patients; mean age: 42.55 ± 11.07 years) or secondary progressive MS course (82 patients; mean age: 42.12 ± 11.95 years) groups. Clinical characteristics of MS patients’ group are shown in supplementary, Table S1.

The total control group was represented by the two independent sample sets: the first, consisting of DNA samples from 15 healthy individuals, was obtained on the basis of the Latvian Center for Marine Medicine, Vecmilgravis Hospital, and the second, 193 DNA samples, referred to the Genome Database of Latvian Population, Latvian Biomedical Research and Study Center (http://biomed.lu.lv/gene/). No significant differences in rs9275596 genetic diversity were found between the first and second Latvian population sample sets, which allowed the data to be grouped for a total control group of 208 healthy individuals (122 females; mean age: 46.37 ± 5.86 years and 86 males; mean age: 53.27 ± 8.82 years) to be used in disease association analysis. The common control group was represented by individuals without accompanying diagnosis of autoimmune and/or cardiovascular, type 2 diabetes mellitus (T2DM), obesity, or any other inflammatory diseases. All subjects are an admixture of representatives of non-Baltic ethnic groups of Riga, forming some “average” genotype for North-Eastern Europe. Sampling was also based on the lack of family ties between patients and representatives from a healthy control group.

The study was performed according to the Declaration of Helsinki, the study protocol was approved by the Central Medical Ethics Committee of Latvia, and informed consent was obtained from all participants of the study.

### 2.2. DNA Extraction and Genotyping

Genomic DNA was extracted from nucleated blood cells using a kit for genomic DNA extraction (Thermo Scientific, Waltham, MA, USA). Basic PCR was performed with DreamTaq polymerase (Thermo Scientific) using the following parameters: 94 °C for 5 min, then 35–40 cycles of 94 °C for 45 s, appropriate annealing temperature (60 °C) for 45 s, 72 °C for 45 s and a final extension step at 72 °C for 7 min.

The SNP rs9275596 C/T (6:32713854) was genotyped by restriction fragment length polymorphism (RFLP) analysis. Oligonucleotide primers were designed using an online Primer-BLAST tool (http://www.ncbi.nlm.nih.gov/tools/primer-blast/): forward, 5′ TTGATGGTTCCACCACAGCA 3′, and reverse, 5′ ATCAGCCAGAGACGAAAGTG 3′. The PCR products (632bp) were digested in a total volume of 10 μL using *Eco57I* (5 U/μL, Thermo Scientific, USA) restriction enzyme.

Quality and quantity of DNA were determined using agarose gel electrophoresis and spectrophotometry. For quality control, 16 randomly chosen samples were genotyped in duplicate in different experiments. The concordance of the genotyping was 100%. Genotyping data were verified by direct sequencing of the corresponding DNA fragments in both directions using the Applied Biosystems 3130*xl* Genetic Analyzer. Loci description and nucleotide numbering is given according to the recommended nomenclature system (http://www.genomic.unimelb.edu.au/mdi/mutnomen/recs.html). The chromosome 6 GRCh38.p12 assembly (NCBI reference sequence: NC_000006.12) sequence information was used for loci description.

### 2.3. Data Management and Analysis

Single locus genotypes and allele frequencies were estimated by direct gene counting. Deviation from the Hardy–Weinberg equilibrium (HWE) and differences between case and control groups in allele and genotype frequencies were evaluated by χ2 of Fisher exact test using IBM SPSS Statistic v.25 (IBM Corp. Released 2017. IBM SPSS Statistics for Windows, Version 25.0. Armonk, NY: IBM Corp, https://www-01.ibm.com/support/docview.wss?uid=swg21476197). Genetic models for investigated locus were designed using different contingency tables and their relationships to the underlying genetic model [25]. Contingency tables were 2 × 3 for the AA, AB, BB for the AA, AB, BB genotypes in the general model; 2 × 2 for the AA, AB + BB and AA + AB, BB, and AB, AA + BB genotypes in the dominant, recessive, and over dominant models, respectively and A and B alleles in the multiplicative model where A is the major allele and B is the minor allele. Using an additive model, the AA, AB, and BB genotype distribution were analyzed using the Cochrane-Armitage test for trend. An odds ratio (OR) of more than two (2) and less than 0.5 was considered to be clinically significant [26]. Sex-specific stratification was performed in MS and control groups.

### 2.4. SNP Functional Analysis in Silico

An eventual functional significance of the SNPs showing evidence of association was analyzed in silico on sequence similarity to transcription factor binding sites (TFBSs) using Genomatix software, MatInspector, Release 7.4 online tool [27] (www.genomatix.de). Only parameters with core/matrix similarity of 1.00/0.85 or more were taken into account. DNA secondary structures were predicted using the Mfold web server [28] (www.bioinfo.rpi.edu/zukerm/cgi-bin/rna-index.cgi). Folding was simulated at 37 °C and with 20 mM Na^+^ and 1.5 mM Mg^++^ for Intracellular or/and 145 mM Na^+^ and 0.5 mM Mg^++^ for Extracellular [29]. If various similar structures were obtained, structures with the highest negative free energy were considered to be representative.

Splicing signals were predicted by Human Splicing Finder Version 3.1 [30] (http://www.umd.be/HSF3/HSF.shtm) with standard threshold values for branch point, donor and acceptor splice sites, enhancer, silencer and other splicing motifs. Sequence similarity to Transfer RNA Genes (tRNA) was analyzed by tRNAscan-SE 2.0 tool [31] (http://lowelab.ucsc.edu/tRNAscan-SE/), and microRNA targets prediction was done using miRBase [32] (http://www.mirbase.org/search.shtml). The Matrix Catch2.7 algorithm was used for potential composite elements (CEs) for transcription factors (TFs) in any DNA sequence [33] (http://gene-regulation.com/cgi-bin/pub/programs/mcatch/MatrixCatch.pl).

## 3. Results

### 3.1. Polymorphism Discovery and Genetic Diversity

In both MS and Latvian population cohorts, the SNP rs9275596 genotyping call rate was 100%, and the marker was found to be in the HWE (*p* > 0.05). Allele frequencies of this SNP were found to be similar between female and male groups in controls and cases (Table 1).

rs9275596 showed statistically significant association with the MS main group and in the female cohort for rare alleles (*p* < 0.001; OR = 1.57, 95% confidence interval, CI [1.20–2.06] and *p* < 0.01; OR = 1.68, 95% CI [1.19–2.37], respectively), in the additive model. Genotypes including rare allele C as a risk factor were identified as MS risk factors in the disease common cohort (*p* < 0.002, in dominant model) and in females (OR = 2.24, 95% CI [1.41–3.57]) using the recessive model. Homozygous genotypes (CC) with rare alleles included were identified as disease susceptible (OR = 2.41, 95% CI [1.01–5.72], in the dominant model) in the male cohort.

### 3.2. Eventual Functional Significance of the SNPs’ Allelic Variants

No motifs were determined for generating tRNA and microRNA targets in the presence of genetic variations of rs9275596 (data not shown), but both major and minor alleles potentially assist in sequence affinity to TFs.

**Figure 1:**
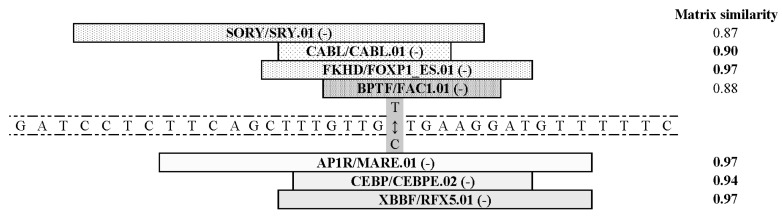
Consequences of the rs9275596 (6:32713854) nucleotide substitutions on functional potential of corresponding genomic region. Localization of transcription factors (TFs) on positive and negative DNA strands are indicated by (+) and (-), respectively. The TFs’ family and matrix names are separated by symbol of division and given according to MatInspector, Release 7.4 online tool at www.genomatix.de.SORY/SRY.01—Sex-determining region Y gene product; CABL/CABL.01—Multifunctional c-Ablsrc type tyrosine kinase; FKHD/FOXP1_ES.01—Alternative splicing variant of FOXP1, activated in embryonic stem cells ESCs; FKHD/FOXP1_ES.01—Alternative splicing variant of FOXP1, activated in ESCs); BPTF/FAC1.01—Fetal Alz-50 clone 1 (FAC1); BPTF/FAC1.01—Fetal Alz-50 clone 1 (FAC1); AP1R/MARE.01—Maf response elements, half sites; CEBP/CEBPE.02—CCAAT/enhancer binding protein (C/EBP), epsilon; XBBF/RFX5.01—Regulatory factor X, 5 (influences HLA class II expression).

**Figure 2:**
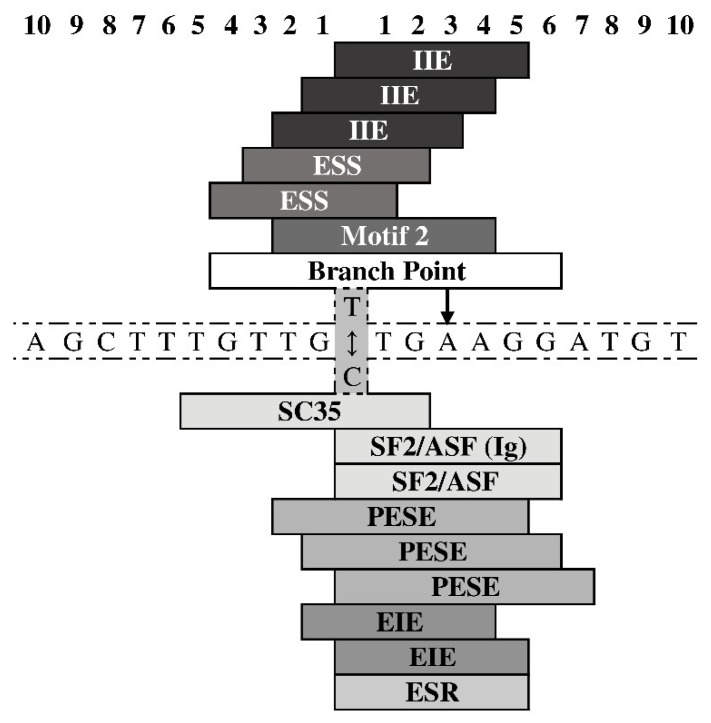
Consequences of the rs9275596 nucleotide substitutions on functional potential of corresponding genomic regions in position near (6:32713854). Splicing enhancer and silencer motifs were analyzed with Human Splicing Finder Version 2.4 [30]. Branch point—potential branch point indicated by an arrow; EIS and IIE—exon- and intron-identity elements, respectively; ESS—exonic splicing silencers; Motif2—Silencer motif; Exonic splicing enhancers: SC35—Serine/arginine-rich splicing factor; SF2/ASF—pre-mRNA-splicing factor/alternative splicing factor; PESE—putative octamer exonic splicing enhancers; ESR—Exonic Splicing Regulatory.

Figure 1 and Figure 2, respectively, summarize results of the in silico analysis of the functional significance of allele substitutions evaluated on the eventual sequence affinity to TFs and splicing signals similarity.

Substitution for rare allele C potentially assists in creation of binding sites (BSs) in proteins of the AP1R, CEBP, and XBBF families, but Major allele T assists to sequence the creation of BSs to TFs of SORY, CABL, FKHD and BPTF families (Figure 1).

The rs2295827 major and ancestral allele T could generate an additional branch point. Both alleles could potentially change the sequence similarity to a number of splicing enhancers and/or silencers (Figure 2).

**Figure 3:**
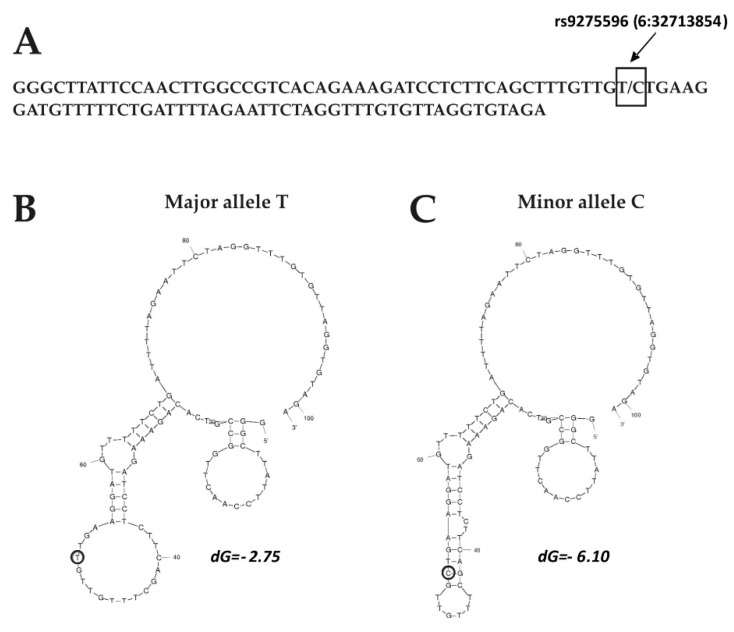
Impact of the single nucleotide polymorphism (SNP) rs9275596 on the DNA secondary structure. (**A**) Sequence included nucleotide variation T/C (6:32713854). SNP is indicated by an arrow and boxed; DNA secondary structures for (**B**) T and (**C**) C alleles. SNP is indicated by arrows; dG, negative energy.

The impact of the SNP rs9275596 on the DNA secondary structure was revealed; nucleotide transition from T to C could generate the changes of the hairpin structure that could finally lead to thermodynamically more stable DNA secondary structures (Figure 3).

## 4. Discussion

**Figure 4:**
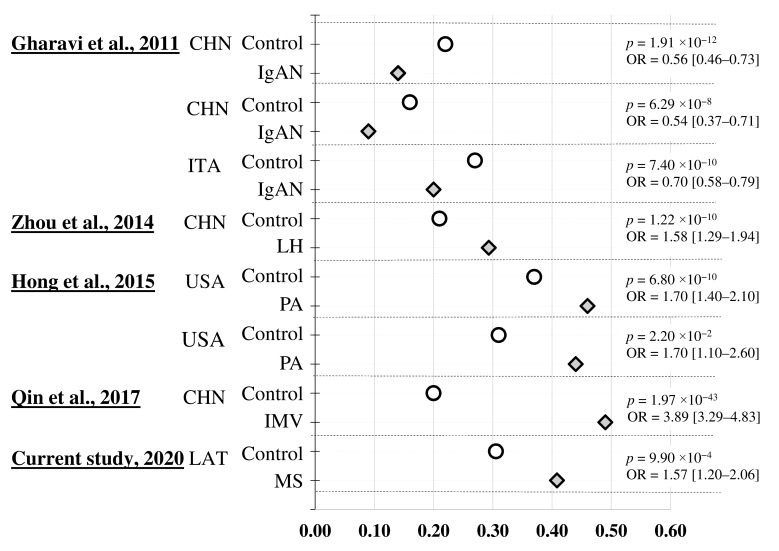
Associations of rs9275596 with particular effects in different ethnic groups. minor allele frequency (MAF) distribution controls and cases in population cohorts. CHN, Chinese Northern Han; ITA, Italian origin; USA, United States of America; LAT, Latvians; IgAN, immunoglobulin A nephropathy; LH, Lupus nephritis; PA, peanut allergy; IMN, idiopathic membranous nephropathy; MS, multiple sclerosis. MAF frequencies in population controls are indicated by white circles; diseases cohorts are represented by gray rhombuses. *p* value and OR of association are given in accordance with the data from the publications.

In the current study, we investigated intergenic SNP rs9275596 (chr6:32713854; RCh38.p12) located in the MHC region linked to MS [11,34,35]. In the Latvian population, rs9275596 rare allele (C) was identified as a risk factor in the MS common cohort (*p* < 0.001) and appears to be disease-susceptible in the female group (*p* < 0.01, Figure 4). Sex-specific differences in incidence, prevalence, and severity are well-known features of autoimmune disease epidemiology [36], including MS [2]. Stratification of the MS cohort in the Latvian population by sex revealed significant differences in alleles (*p <* 0.01, multiplicative model) and genotypes frequency (*p <* 0.001, recessive model; Table 1). It is noteworthy that, according to the results of testing associations using various genetic models (Table 1), it can be assumed that rs9275596 heterozygotes, as possible risk factors, can have a greater impact on the predisposition to the MS in Latvian female, but the homozygotes of rare alleles were more susceptible to diseases in the male group.

The MS-associated SNP discovered in our study potentially could be themselves primarily susceptible to disease or linked with other primary genetic variations linked to disease. It appears that both scenarios are possible. Previously, the interaction between the rs9275596 and *RUNX1*-rs1542876 locus was revealed, previously reported to be related to rheumatoid arthritis [37], systemic lupus erythematosus [38], and psoriasis [39]. This polymorphism was previously discovered in association with increased susceptibility to immunoglobulin A nephropathy (IgA) in Chinese patients [22] (Figure 4). It was significantly associated with macroscopic hematuria of IgA patients from the Western Chinese Han population [40]; this polymorphism’s minor variation had the exact opposite effect on the pathogenesis of IMN and IgAN, indicating a completely different role for this SNP for two types of primary glomerulonephritis [41] (Figure 4).

rs9275596 was identified as a Peanut Allergy (PA)-specific locus in a GWAS in participants of European ancestry; the result obtained was confirmed in a replication study of independent samples from the same cohort [42] (Figure 4). This SNP achieves genome-wide significance in IgAN study with a consistent effect size in Chinese and European ancestry [15]; it was found to be a top significant marker (*p* = 1.41 × 10^−8^) and showed a cumulative genetic risk effect for multiple sclerosis from GWAS association data [35]. Finally, these results demonstrated that the SNP rs9275596 is shared with other immune-mediated diseases, suggesting that clinically distinct autoimmune phenotypes may share common genetic susceptibility factors.

In our study, we have attempted to evaluate the rs9275596 genetic variations’ possible impact on the genes’ transcriptional regulation. To reach this goal, we constructed and compared the allele-specific TFBS patterns and analyzed the potential change in sequence similarity with a number of regulatory factors and DNA secondary structures.

It was found that rs9275596 locus structure variations are functionally active with respect to splicing due to the fact that they could influence DNA secondary structure in sequence regions of splice site recognition [43]. We can assume that such a perturbation of the secondary structure of DNA can potentially affect the interaction of a sequence with various regulatory proteins with the efficiency of transcription and splicing of a sequence variant.

This may have a special role in the sequence interaction with other regulatory proteins, particularly with SORY family proteins. The nucleotide T defines the affinity to the SRY.01 (Sex-determining region Y gene product) that binds DNA sequences, specifically in the minor groove, resulting in substantial DNA bending [44]. The CABL family protein acts as a signaling promoter or as a signaling inhibitor [45], thus functioning as a “switch modulator”. In turn, nucleotide substitution C creates binding sites to the CEBPE protein, important for monocyte and granulocyte development, and is essential for a normal terminal differentiation of committed granulocyte progenitor cells [46] and to XBBF family regulatory factor, which influences HLA class II genes expression by the promoter’s activation [47]. In a previous study, rs9275596 was identified as a quantitative methylation locus for the *HLA-DRB1* and *HLA-DQB1* genes. Thus, it may regulate the expression levels of these genes and subsequently may partly mediate the genetic risk of MS [42]. Therefore, we can assume that the presence of many regulatory elements in the SNP localization region provides combinatorial control of distal gene regulation. Summarizing the mentioned results, we suggest that the nucleotide substitutions we have studied may significantly modulate the transcription of genes related to the major histocompatibility complex (MHC) and gene network in response to the inflammation and other environmental stimuli and influence the multiple sclerosis susceptibility.

Therefore, all of the above types of associations revealed and data on functional significance of allele substitutions are in good agreement between themselves and provide evidence that (1) variations at the Chr. 6p21 region rs9275596 locus could assist MS susceptibility; (2) the rs9275596 genetic variants could be suggested as MS sex-specific risk factors; (3) nucleotide substitutions affect the potential of encompassing sequences to create TFBSs and different regulatory elements and could generate the changes in DNA secondary structure.

Our study has some limitations; the interactions of the rs9275596 locus were not investigated with respect to the clinical characteristics of MS. Interactions in the analysis of the clinical characteristics and prognosis may be difficult due to limited events.

It should be mentioned that, despite the rather small number (273/208 of cases/controls), this study can be considered as representative for the small Latvian population (<1.9 million). Keeping in mind that MS tends to affect females more than males, we have applied stratification by sex. Due to the small subgroups, we sometimes could not reach significance. However, significance was achieved and reveals the trend of the association with the disease; our results appear to predict the common association trends for larger sample groups.

To our best knowledge, we have reported for the first time that rs9275596 may be a predictor of MS prognosis. In future studies, we intend to analyze the interactions of this polymorphism with respect to the clinical characteristics of multiple sclerosis and specific treatments, such as interferon therapy. Considering that MS affects people in all countries and all ethnic groups, we also take into account that the prevalence of this pathology varies significantly in different regions [1]. In order to understand the origin of MS and to manage it effectively, it is necessary to understand the reasons causing these geographical differences. Thus, replication in the data sets of the independent population is necessary to verify the results of the above associations obtained in the Latvian population.

## 5. Conclusions

Our findings provide evidence that rs9275596 locus variations in the chr6:32713854 (GRCh38.p12) are associated with multiple sclerosis in the Latvian population, they could play an important role in MS and other immune-mediated pathologies in both Caucasians and Asians, and could be suggested as sex-specific genetic risk factors. As follows from our data, rs9275596 may represent a potential independent MS predisposing marker.

## Figures and Tables

**Table 1:** Distribution of alleles and genotypes of rs9275596 polymorphism and data on association with multiple sclerosis in Latvian population.

MAF or Genotype	Distribution of Allele and Genotypes					Data on Association			
	Groups	MS *		Controls ^		Genetic Model	Group	*p*	OR [95% CI]
		*n*	%	*n*	%				
C ^♦^	Total	223	40.84	127	30.53	C vs. T	Total	0.00099 **	1.57 [1.20–2.06]
	Females	156	40.84	71	29.10		Females	0.0029 *	1.68 [1.19–2.37]
	Males	67	40.85	56	32.56		Males	n.s.	—
TT	Total	96	35.16	103	49.52	TC + CC vs. TT	Total	0.0015 *	1.81 [1.25–2.63]
	Females	63	32.98	64	52.46		Females	0.00062 **	2.24 [1.41–3.57]
	Males	33	40.24	39	45.35		Males	n.s.	—
TC	Total	131	47.99	83	39.90	TC vs. TT + CC	Total	n.s. (0.08)	—
	Females	100	52.36	45	36.89		Females	0.0074 *	1.88 [1.18–2.99]
	Males	31	37.80	38	44.19		Males	n.s.	—
CC	Total	46	16.85	22	10.58	CC vs. TT + TC	Total	0.050 *	1.71 [0.99–2.95]
	Females	28	14.66	13	10.66		Females	n.s.	—
	Males	18	21.95	9	10.47		Males	0.043 *	2.41 [1.01–5.72]

* The number of patients in MS groups: Total = 273; Females = 191; Males = 82; ^ the number of patients in the control groups: Total = 208; Females = 122; Males = 86. The number of genotypes in the groups is equal to the number of samples, (^♦^) the number of alleles is represented by twice the number of genotypes, MAF—minor allele frequency; *p*—probability calculated by χ^2^ or Pearson exact tests; OR [95% CI]—odds ratio with 95% confidential interval, indicated when n/s (no statistical result or *p* < 0.05). Nominal and moderate statistical significance corresponds to * *p* < 0.05 and ** *p* < 0.001.

## Supplementary Materials

The following are available online at https://www.mdpi.com/1010-660X/56/4/154/s1, Table S1: Clinical characteristics of group of patients of Multiple sclerosis (MS).
