# Supplementary material for: An Intergenic rs9275596 Polymorphism on Chr. 6p21 Is Associated with Multiple Sclerosis in Latvians
Source: Medicina (Kaunas). 2020 Mar 31;56(4):154. doi: 10.3390/medicina56040154 (PMC7230508; doi:10.3390/medicina56040154)
Supplement: Supplementary file 1 [file medicina-56-00154-s001.pdf]

Supplementary Material

Table S1 Clinical characteristics of a group of patients with multiple sclerosis (MS)

Factor	Patients - All (n = 273)	Patients – women (n = 191)	Patients – men (n = 82)
Age of illness (years) in time of:			
1st symptoms	28.99 ± 9.11	28.54 ± 8.88	30.05 ± 9.62
1st visit and registration of MS	35.73 ± 10.56	34.50 ± 10.52	36.15 ± 10.52
Disease course (years)			
from 1st symptoms	13.43 ± 9.77	14.01 ± 10.04	12.07 ± 9.03
from 1st visit	7.02 ± 4.38	7.13 ± 4.50	6.75 ± 4.11
Other autoimmune diseases (yes number; %)	35 (12.82)	27 (14.13)	8 (9.75)
Expanded Disability Status Scale (EDSS; points)	3.65 ± 1.71	3.72 ± 1.75	3.50 ± 1.61
MS phenotype:			
Relapsing-remitting (number; %)	185 (67.77)	128 (67.02)	57 (69.51)
Secondary progressive (number; %)	88 (32.23)	63 (32.98)	25 (30.49)
Laboratory parameters:			
Immunoglobulin G (IgG)	11.68 ± 2.57	11.87 ± 2.47	11.24 ± 2.74
Immunoglobulin A (IgA)	2.19 ± 0.80	2.13 ± 0.79	2.32 ± 0.80
Immunoglobulin M (IgM)	1.63 ± 0.72	1.71 ± 0.77	1.46 ± 0.56
Cluster of differentiation 3 (CD3)	1.43 ± 0.41	1.43 ± 0.40	1.44 ± 0.41
Cluster of differentiation 4 (CD4)	0.90 ± 0.28	0.90 ± 0.26	0.88 ± 0.33
Cluster of differentiation 8 (CD8)	0.54 ± 0.22	0.53 ± 0.21	0.56 ± 0.23
Age (years)	42.42 ± 11.31	42.55 ± 11.07	42.12 ± 11.95
Special medicament therapy (yes number; %)	193 (70.70)	136 (71.20)	57 (69.51)
